# Species distribution models and empirical test: Comparing predictions with well‐understood geographical distribution of *Bothrops alternatus* in Argentina

**DOI:** 10.1002/ece3.4517

**Published:** 2018-10-02

**Authors:** Juan A. Sarquis, Maximiliano A. Cristaldi, Vanesa Arzamendia, Gisela Bellini, Alejandro R. Giraudo

**Affiliations:** ^1^ Instituto Nacional de Limnología (CONICET – UNL) Ciudad Universitaria Santa Fe Argentina; ^2^ Facultad de Humanidades y Ciencias (FHUC – UNL) Ciudad Universitaria Santa Fe Argentina

**Keywords:** experts maps, fuzzy global matching, niche modeling, similarity, snake

## Abstract

Species distribution models (SDMs) estimate the geographical distribution of species although with several limitations due to sources of inaccuracy and biases. Empirical tests arose as the most important steps in scientific knowledge to assess the efficiency of model predictions, which are poorly rigorous in SDMs. A good approach to the empirical distribution (ED) of a species can be obtained from comprehensive empirical knowledge, that is, well‐understood distributions gathered from large amount of data generated with appropriate spatial and temporal samples coverage. The aims of this study were to (a) compare different SDMs predictions with an ED; and (b) evaluate if fuzzy global matching (FGM) could be used as an index to compare SDMs predictions and ED. Six algorithms with 5 and 20 variables were used to assess their accuracy in predicting the ED of the venomous snake *Bothrops alternatus* (Viperidae). Its entire distribution is known, thanks to thorough field surveys across Argentina, with 1,767 records. ED was compared with SDMs predictions using Map Comparison Kit. SDMs predictions showed important biases in all methods used, from 70% sub‐estimation to 40% over‐estimation of ED. BIOCLIM predicted ≈31% of *B. alternatus* ED. DOMAIN predicted 99% of ED, but over‐estimated 40% of the area. GLM with five variables calculated 75% of ED, while Genetic Algorithm for Rule‐set Prediction showed ≈60% of ED; the last two presenting overpredictions in areas with favorable climatic conditions but not inhabited by the species. MaxEnt and RF were the only methods to detect isolated populations in the southern distribution of *B. alternatus*. Although SDMs proved useful in making predictions about species distribution, predictions need validation with expert maps knowledge and ED. Moreover, FGM showed a good performance as an index with values similar to True Skill Statistic, so that it could be used to relate ED and SDMs predictions.

## 1 INTRODUCTION

In recent decades, increased use of GIS and technical tools that quantify species–environment relationships has encouraged the development of algorithms to predict the spatial distribution of species, called species distribution models (SDMs) (Elith & Leathwick, 2013; Guisan & Zimmermann, 2005). SDMs relate species occurrence data with a set of variables selected under the assumption that they could be related to the distribution of the species (Guisan & Zimmermann, 2005). They are being increasingly used to assess conservation applications and climate change studies, and predict both ecological ranges and the potential of invasive species and explicit predictions about species environmental suitability (Bosso et al., 2013; Chen, Zhang, Jiang, Nielsen, & He, 2011; Law et al., 2004). SDMs are favored by an increased access to public biodiversity (e.g., Biodiversity Information System for Europe, Global Biodiversity Information Facility and Sistema de Información de Biodiversidad) and environmental databases (e.g., Data Service and Information, Global Environmental Database, WorldClim), being also a promising tool to fill knowledge gaps in species distributions (Guillera‐Arroita et al., 2005; Guisan et al., 2012). The lack of distributional data, the so‐called Wallacean shortfall, stems from geographical biases, which often result in maps of observed biodiversity closely resembling maps of survey effort (Hortal, Borges, & Gaspar, 2007; Hortal et al., 2008; Lomolino, 2014; Whittaker et al., 2005). However, unbiased species distribution information is important to make robust conservation management decisions (Guisan et al., 2012).

Although SDMs were assessed with different thresholds, sample sizes, variables, and background (Barbet‐Massin, Jiguet, Albert, & Thuiller, 2012; Bucklin et al., 1991; Elith et al., 2009; Guisan & Thuiller, 2003; Guisan & Zimmermann, 2005; Jiménez‐Valverde, Lobo, & Hortal, 2017; Peterson et al., 2007; Qiao, Peterson, & Soberon, 2009; Saupe et al., 2014), they present several limitations listed in Elith et al. (2009) and Mateo, Felicísimo, and Muñoz (2011). While many studies take into account these limitations (Araújo & Luoto, 2007; Elith et al., 2009; Fitzpatrick, Weltzin, Sanders, & Dunn, 2001; Guisan et al., 2000; Jarnevich, Stohlgren, Kumar, Morisette, & Holcombe, 2003; Oliveira et al., 2015; Rojas‐Soto, Mart, & Navarro‐sig, 2014; Rojas‐Soto, Sosa, & Ornelas, 2018; Tsoar, Allouche, Steinitz, Rotem, & Kadmon, 2003; Varela et al., 2015), few compare model predictions with well‐known distributions of species (e.g., Duan, Kong, Huang, Fan, & Wang, 2006; Elith et al., 2009) or virtual species with well known niches (e.g., Qiao et al., 2009; Saupe et al., 2014), these last studies being really important steps to assess the accuracy of different SDMs predictions. In this context, Soberón and Peterson (2008) proposed a formal basis to clarify the use of techniques towards estimating ecological niches. Ecological niches could be represented by the BAM diagram (biotic; abiotic; movement) (See figure 1 of Soberón & Peterson, 1999). This diagram combines three factors: biotic (B) and abiotic (A) factors, as well as dispersed accessible regions (M), whose intersections represent the geographic space occupied by the species (Soberón, 2007; Soberón & Nakamura, 2007) and the intersection of A + B represents the potential distribution (Soberón & Peterson, 2008), being one of the most expected applications in SDMs studies (e.g., Urbina‐Cardona & Loyola, 2008). As in Saupe et al. (2014), we could use BAM diagram to distinguish between two conceptual frameworks in this field, one to estimate the occupied area (B + A + M) and the other to estimate the potential distribution (B + A). The first requires information about favorable conditions and factors that restrict its spread (biotic and geographic factors) to avoid over predictions (Peterson et al., 1999). The other needs only the favorable conditions, being the potential distribution areas (Saupe et al., 2014; Soberón & Peterson, 2008).

Nonetheless, studies that provide techniques to improve SDMs predictions in relation to expert maps are needed, as in Merow, Wilson, and Jetz (2010) where they sought to determine if expert maps can help reduce biased extrapolation in SDMs prediction. In a similar way, most of the accuracy measures from the confusion matrix (Barbosa, Real, Muñoz, & Brown, 2015; Fielding & Bell, 2018) and indices (e.g., area under the curve—AUC‐ROC; Akaike information criterion, see Guisan & Thuiller, 2003) used for this purpose does not provide a comparison of the model with the empirical distribution (ED) of the species (Loiselle et al., 2011). These last authors pointed out the importance of validating models with independent data, and warned that failure to include independent model validation, especially in cases where training points are limited, may potentially lead to serious errors in conservation decision‐making. In this sense, one of the most important steps in scientific knowledge is carrying out empirical tests to assess the efficiency of model predictions. A good approach to the “ED” of a species can be obtained from empirical knowledge (Merow et al., 2010). Expert maps, in fact, are usually an excellent resource for delimiting the broad areas outside which a species is not expected to occur (Merow et al., 2010) and, in the case of well‐understood distributions with large amount of data generated by specialists and appropriate spatial and temporal unbiased sample coverage, they could be considered the best approach to define empirical geographical distributions (Loiselle et al., 2011; Merow et al., 2006). On the other hand, Power, Simms, and White (2012) and White (2006) have previously demonstrated that the fuzzy global matching (thereafter FGM) function used as comparison tool provide a good interpretation to compare empirical maps and models prediction. The FGM function offers a visual representation of where differences have occurred between two maps. Recent findings as Barbosa and Real (2013) highlighted several advantages of fuzzy logic over as a tool to compare models predictions, such as the possibility to combine multiple species models. In this context, the aims of this study were to (a) compare different SDMs predictions with an ED of *Bothrops alternatus*; and (b) evaluate if FGM could be used as an index to compare SDMs predictions with ED. As a model for this study, we selected *B. alternatus*, a poisonous snake species with public health importance. Thorough and continuous efforts have been made for decades to perform unbiased samplings throughout its distribution area in Argentina (e.g., Arzamendia & Giraudo, 2012; Bellini, Giraudo, Arzamendia, & Etchepare, 2017; Giraudo, 2018; Giraudo & Arzamendia, 2006; Giraudo et al., 2002; Nori, Carrasco, & Leynaud, 2001). Besides, *B. alternatus*: (a) presents a well‐understood distribution; (b) is easily detectable (it lives in relatively wet mesophilic open areas including grasslands, savannas, wetlands, and open forests in the Espinal, where exhaustive surveys are possible; (c) has a large and conspicuous size and is relatively abundant throughout its spatial distribution); (d) offers plenty of information about its natural history such as diet, habitat use, and reproduction (Bellini et al., 2017; Giraudo, 2008; Giraudo et al., 2002; Scrocchi, Moreta, & Kretzschmar, 2009); (e) presents peculiarities in its distribution which constitute real challenges in modeling; for example, it has not been found in humid forests to the northeast of its distribution (Giraudo, 2018) and two disjunctive and isolated populations occur in coastal areas and the Pampean hills in Buenos Aires province (South distribution).

## 2 MATERIALS AND METHODS

### 2.1 Study area and data

**Figure 1:**
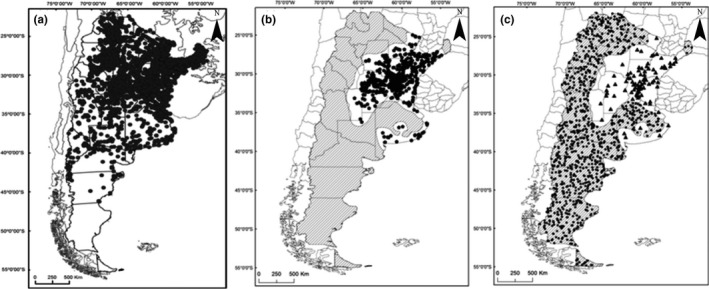
Records of the snakes that inhabit Argentina. (a) 21,032 records of Argentinean snakes, obtained in 382 field works between 1989 and 2017 throughout the country plus museums and literature data. (b) Black points correspond to 1,767 georeferenced records of *B. alternatus*, while the black lines represent the area delimited by us where *B. alternatus* does not occur. (c) Subset of 100 randomly selected presence records (training data) and 1,000 randomly created absences in the area where the species does not occur

Of 21,032 records of Argentinean snakes in our database (Arzamendia & Giraudo, 2004, 2012; Giraudo & Arzamendia, 2015) (Figure 1a), we extracted 1,767 occurrence data of *B. alternatus* (Figure 1b). These data were recorded in 385 of our own field works, in different seasons and throughout the country, between 1989 and 2017, mainly in poorly sampled areas (gap areas) in order to complement biases in the database. In addition, we revised museum collections to confirm taxonomic identification and obtained reliable georeferenced data from the scientific literature, both tasks dating back to the beginning of the 20th century.

We defined the ED of *B. alternatus* (Figure 1b) mainly based on both collected data with intensive spatial and temporal sample coverage and our knowledge (Bellini et al., 2017; Giraudo, 2018; Giraudo & Arzamendia, 2006; Giraudo et al., 2002) as well as by delimiting areas inhabited and not inhabited by the species. We included some areas without data in the inhabited territory of *B. alternatus* because we know that, they can be occupied by the species (Hortal et al., 2008). More specifically, ED was delimited following the peripheral presences, leaving a buffer distance of 30 km for similar environmental areas and 5 km when the climatic conditions changed abruptly (e.g., a mountain; like in the south of *B. alternatus* distribution). So, we combined our knowledge to generate the ED and the presence data following Merow et al. (2006). This ED was compared with the predictions produced by each model. For *B. alternatus*, some environmental areas present suboptimal conditions that change throughout the country, comprising large areas in the North and smaller areas in the South, where some disjunctive populations inhabit the Tandil and Ventana hills in Buenos Aires province.

The minimum allowed distance (5 km) function in ArcGis 10.1 was used to randomly select a total of 350 occurrences. As a sample size of <70 observations reduces model performance (Kadmon, Farber, & Danin, 2010), and increasing sample size decreases the variability in predictive accuracy (Wisz et al., 2008), we selected 100 presences for calibration and the evaluation process (Figure 1c). In this way, we evaluated SDM performances with a small sample size, a situation pointed out in numerous studies (e.g., Barbet‐Massin et al., 2012; Wisz et al., 2014). Although random selection for the presence/absence of data does not allow to obtain independent samples and therefore can overfit the calibration or training data (Araújo, Thuiller, Williams, & Reginster, 2009), this is not considered a problem if the goal is to describe a pattern and simultaneously reduce false‐negatives (Araújo & Guisan, 2006). If SDMs are intended to be used for conservation planning, verification becomes an approved method to test whether an SDM performs as intended (Raes & ter Steege, 1999).

### 2.2 Environmental predictors

We used 19 climatic variables taken from WorldClim (http://www.worldclim.org/bioclim) and one topographical variable (altitude with a 1 km‐ resolution) taken from R‐package Raster (http://srtm.csi.cgiar.org/ in Hijmans, Cameron, Parra, Jones, & Jarvis, 2015; Hijmans et al., 2015). We used only environmental predictors because evidence (historical and modern) demonstrates that climatic variables play a primary role in shaping species’ distributions (Fourcade, Besnard, & Secondi, 2012). We chose this resolution because it represents more effectively the variability of the species in the 20 variables used for the analysis. Soberón and Nakamura (2007) said that grid resolution should be established by biological considerations of the size, mobility, and ecology of the species. In this case, *B. alternatus*'s home‐range is usually not very wide and there are areas within its distribution (the Pampean hills in Buenos Aires province) where the species presents environmental differences in the presence/absence at 1 km resolution in its distribution (Bellini et al., 2017; Giraudo, 2018; Giraudo & Arzamendia, 2006; Giraudo et al., 2002; Scrocchi et al., 2009). We performed a Spearman correlation test in order to get the least collinear predictor subset using Infostat 5.1 (Di Rienzo et al., 2017). We chose variables with a correlation value lower than 0.7 and confirmed the selection of the variables with the knowledge about the natural history of the species (Bellini et al., 2017; Giraudo, 2008; Giraudo et al., 2002; Scrocchi et al., 2009) (Supporting Information Appendix S1). This to ensure a controlled collinearity between predictors and to avoid biased results (Acevedo, Jiménez‐Valverde, Lobo, & Real, 2012). We confirmed that the selected predictor variables were related to likely occupied areas rather than potentially suitable areas, thus avoiding the influence of accuracy on SDM predictions (Elith & Leathwick, 2013; Syfert et al., 2006). The modeling process was performed with two sets of predictors (a set of 5 and a set of 20 variables) following previous studies which concluded that some algorithms are more sensitive to collinearity, while others are very restrictive when using more predictor variables (Elith et al., 2009; Stockwell & Peterson, 2016; Wang, Liu, Munroe, Cao, & Biermann, 2016). For the set of 20 variables, we used the 19 climatic variables and altitude (m).

### 2.3 Modeling procedure

We assessed six of the most commonly used modeling methods (Graham & Hijmans, 2006), following Elith et al. (2009), grouped in two types of algorithms. One group includes presence‐only algorithms (e.g., BIOCLIM, DOMAIN). BIOCLIM characterizes sites that are located within the environmental hyper‐space occupied by a species and calculates suitability values across the geographic region in terms of climatic and topographical conditions similar to the hyper‐space (Busby, 1993). DOMAIN uses a point‐to‐point similarity metric to assign a classification value to a candidate site based on the proximity of the environmental space to the most similar record site (Gower distance), resulting in a probabilistic map (Carpenter, Gillison, & Winter, 2017). The second group of algorithms is composed of methods that characterize the background with a sample, such as Genetic Algorithm for Rule‐set Prediction (GARP) and Maximum Entropy (MaxEnt), or that sometimes use pseudo‐absences and/or presence data, like several general lineal models (GLM) regression approaches and Random Forest (RF). GARP uses a genetic algorithm to select a set of rules (e.g., adaptations of regression and range specifications) that best predict species distribution (Stockwell & Peters, 2006). MaxEnt estimates the distribution of maximum entropy constrained in such a way that expected values for predictor variables match their empirical average (Phillips, Anderson, & Schapire, 2008). GLM is based on an assumed relation between the mean of the response variable and the linear combination of the explanatory variables (Guisan, Edwards, & Hastie, 2013). RF is considered an “ensemble learning” method of classification trees, each capable of producing a response when presented with a set of predictor values. Each tree, constructed using a different bootstrap sample of the data, grows to maximum size without pruning, trying to maintain some prediction strength while inducing diversity among trees (Breiman, 2015). We included presence–absence models to compare with presence‐only models because tend to performed better (Elith et al., 2009). BIOCLIM and DOMAIN were implemented with DIVA‐GIS (http://www.diva-gis.org), both using default settings (Busby, 1993; Carpenter et al., 2017). GARP was used choosing the best result subset, as explained in the “Open Modeller” module (Anderson, Lew, & Peterson, 2003). MaxEnt was employed following Phillips et al. (2008). GLM and RF were performed in R (R Core Team, 2005) with R‐package Biomod2 (Thuiller, Georges, Engler, & Breiner, 2008). For those models that needed presence/absence data, we generated 1,000 random absence records outside the distribution area (ED) (Tognelli, Roig‐junent, Marvaldi, Flores, & Lobo, 2006), where the evidence in the last 100 years showed that *B. alternatus* does not presently occur but rather became true absences, which are based on reliable field evidence of nonoccurrence (Figure 1c) (Saupe et al., 2014). Moreover, several studies obtained good performance using pseudo‐absence/absence data outside a predefined region based on a minimum distance to the presence (Barbet‐Massin et al., 2012; Lobo, Jiménez‐Valverde, & Hortal, 2018). This way of generated absence records is recommended when using classification and machine‐learning techniques (Barbet‐Massin et al., 2012). These last authors pointed out that the accuracy increases until an asymptote when the number of presences reached one tenth of the number of absences for GLM and RF.

In spite of knowing the distribution of *B. alternatus*, there is always the possibility of finding individuals in the periphery of their distribution. So, the area in Figure 1c where the species does not occur was outlined considering suboptimal areas with low abundance (Figure 1c). Although large backgrounds are merely informative, it is worth noting that they result in high discriminatory power in model prediction (Acevedo et al., 2012).

We generated only one kind of absence data because the variability arising from each methodological choice regarding the use of absences was lower than that arising from the use of different SDMs (see Barbet‐Massin et al., 2012), especially when at least 100 presence records were sampled. We ran each algorithm with both sets of 5 and 20 variables. Therefore, we had B5 and B20 for Bioclim, D5 and D20 for Domain, G5 and G20 for GARP, M5 and M20 for MaxEnt, GL5 and GL20 for GLM, and RF5 and RF20 for Random Forest.

### 2.4 Validation and evaluation methods

Each prediction was converted into a binary map (presence/absence) using ArcGis 10.1 (ESRI 1997). Although a binary map may lead to unnecessary information loss and hence be detrimental in the context of the intended application (Guillera‐Arroita et al., 2005), our purpose was to compare the output prediction map and the ED of *B. alternatus*. We used the threshold value that optimizes specificity and sensitivity for each model. This has the advantage of giving equal weight to both presence and absence success probability when species presence/absence distribution records are unbalanced (Jiménez‐Valverde & Lobo, 2015).

The ED was compared with the cartographic representation of each prediction (12 in total). This analysis was performed after the modeling process and the transformation to a binary map (categorical maps). We established the similarities of each prediction with the ED of *B. alternatus* (Figure 1), overlapped each prediction with the ED and obtained 12 overlapped maps (Hagen, 2016). We quantified the differences between the ED and the cartographic representation of each prediction using the FGM function in the Map Comparison Kit (MCK) 3.2.3 software (Hagen, 2016; http://www.riks.nl/mck). This compares the overlap of two maps, one considered as “reference” (ED) and the other as “comparison” (the models), and results in an overall similarity value, taking into account the intersection area, the area of agreement/disagreement and the polygon size (White, 2017). This analysis was complemented with the Per Category function, which makes a cell‐by‐cell comparison and provides information about the occurrence of the selected category between both maps (Visser & Nijs, 2012) (Figure 3). We calculated percentage indices to show the proportion of cells correctly and incorrectly predicted by the models. These indices were determined with MCK, using the values obtained from each cell and overlaying each prediction with the actual distribution of *B. alternatus*.

In addition, we carried out a Spearman correlation analysis (*p* < 0.05) between FGM and the precision measurements to find which measures were most related to FGM and, therefore, which measure gave more information about the ED. We used several discrimination indices derived from the confusion matrix, namely sensitivity, specificity (Fielding & Bell, 2018), and the under‐prediction and overprediction rates (UPR and OPR, respectively). The latter rates refer to the proportion of observed presences in the predicted absence area and the proportion of observed/assumed absences in the predicted presence area, respectively (Barbosa et al., 2015). We obtained the ROC curve, that is, AUC index, which represents the probability that the model correctly predicted the observed presences and absences and varies from 0 to 1, 1 being perfect discrimination and 0.5 to 0 implying a discrimination worse than random (Araújo et al., 2009; Elith et al., 2009). One of the greatest advantages of the ROC curve (AUC) is that it is threshold independent (Lobo, Jiménez‐Valverde, & Real, 2018); however, its use and efficiency has been widely criticized (Jiménez‐Valverde, 2012; Lobo et al., 2018), although it continues to be used in the literature (e.g., Ma & Sun, 2016; Taylor, Papeş, & Long, 2005). Other metrics have been proposed to evaluate SDMs (see Hijmans, 2006; Phillips & Elith, 2012), despite this, no measure has succeeded in replacing AUC, which is still being used in more than 80% of SDMs studies (Fourcade et al., 2012). We calculated the true skill statistic (TSS), which does not depend on the prevalence or the sample size (Allouche, Tsoar, & Kadmon, 2006). TSS ranges from −1 to +1, where +1 indicates perfect agreement and values of zero or less indicate a performance no better than random (Allouche et al., 2006). These indices were obtained for both training and test data (Figure 4), values >0.7 being considered good predictive accuracies (Faleiro, Machado, & Loyola, 2007).

## 3 RESULTS

### 3.1 Comparison between models and real distribution

**Figure 2:**
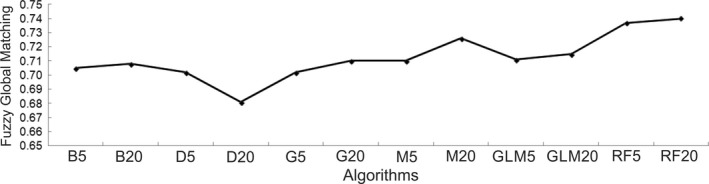
Graphic with fuzzy global matching values for each model compared to the real distribution, where (B5–B20) Bioclim, (D5–D20) Domain, (G5–G20) Garp, (M5–M20) MaxEnt, (GL5–GL20) GLM, and (RF5–RF20) Random Forest

The FGM values were between 0.681 (D20) and 0.740 (RF20) (Figure 2). RF and M20 reached the highest FGM values (0.740 and 0.726, respectively, Figure 2).

**Figure 3:**
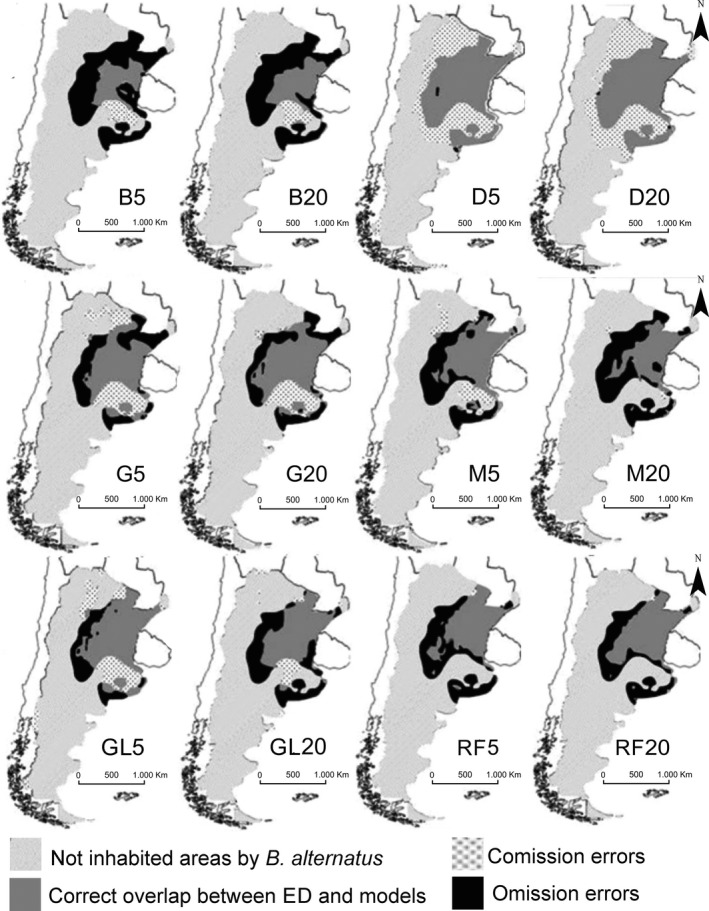
Cell by cell comparison per category between each model (with 5 and 20 climatic variables) and the empirical realized distribution of *Bothrops alternatus*. The light gray area represents areas where *B. alternatus* does not occur (for the model and the real distribution); the dark gray area shows the correct overlap of the model and the real distribution; the black area refers to the empirical realized distribution that is not predicted by the algorithms (omission errors); black points with white background are part of the prediction where *B. alternatus* does not occur (commission errors). Where (B5–B20) Bioclim, (D5–D20) Domain, (G5–G20) Garp, (M5–M20) MaxEnt, (GL5–GL20) GLM, and (RF5–RF20) Random Forest

**Figure 4:**
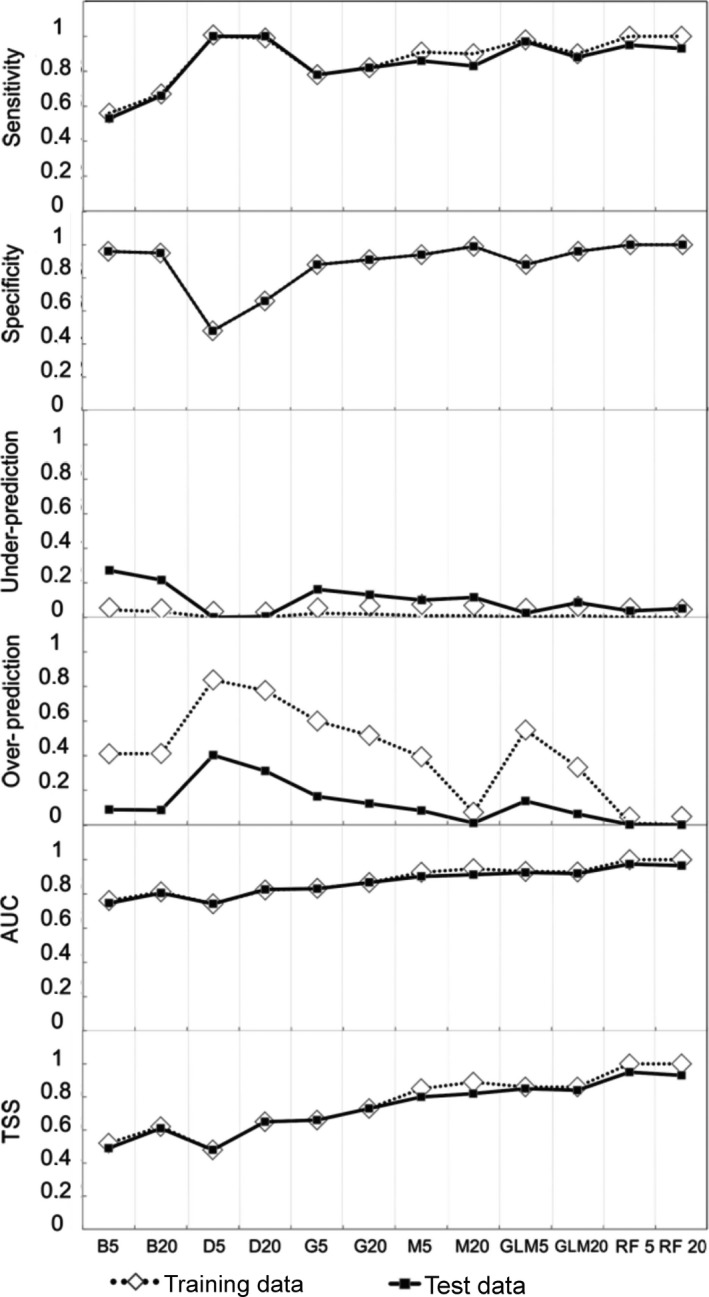
Comparison between the training and test data of the species using the accuracy of sensitivity, specificity, true skill statistic (TSS), area under the curve (AUC), under‐prediction rate, and overprediction rate. Plots showing sensitivity, specificity, Under‐predictions rate, Overpredictions rate, AUC, and TSS for each method of modeling. Diamonds represent values obtained for the training (*N *=* *100), while black squares represent values obtained for the test data (*N *=* *766). Where (B5–B20) Bioclim, (D5–D20) Domain, (G5–G20) Garp, (M5–M20) MaxEnt, (GL5–GL20) GLM, and (RF5–RF20) Random Forest

DOMAIN predicted the highest percentage of the ED (close to 99%), but overestimated almost 40% of the area, while BIOCLIM predicted the lowest proportion of the ED (29%–31%) and showed the highest omission error values (≈70%, Table 1). The rest of the models showed a more balanced trade‐off in overlapping proportions between ED and modeled distribution maps, from intermediate to high overlapping values (43%–75%, Figures 3 and 4). GLM5, for example, predicted 75% of the ED, but it presented a high overprediction rate (Table 1). GARP showed intermediate ED percentages (more than 60%), with relatively poor rates of under‐ and overprediction, but better values than BIOCLIM and DOMAIN. RF predicted 54%–57% of the ED and MaxEnt between 43% and 56%. It is remarkable that the only methods that detected isolated and gap populations in the Pampean hills in Buenos Aires province were M20 and RF5‐20 (see Figures 1 and 3). Moreover, except for B5 and RF5, the rest of the predictions indicated that the same area in Tucumán had high values of suitability (Figure 4, region with white background and black points). We found important differences between the ED of *B. alternatus* and each of the 12 predictions obtained for the species (Figure 3).

**Table 1:** Proportion of cells correctly and incorrectly predicted between each model and the empirical realized distribution of *B. alternatus*

Model types	Sensitivity	Specificity	Overprediction rate	Under‐prediction rate	Total cells detected
B20	34.51	59.34	9.36	65.45	31.32
B5	29.52	64.21	8.82	70.45	26.94
D20	99.61	0.18	41.74	0.31	58.01
D5	99.43	0.35	39.45	0.59	60.23
G20	65.42	28.64	17.16	34.52	54.21
G5	62.71	28.92	22.38	37.23	48.74
GLM20	57.43	38.71	8.81	42.52	52.36
GLM5	75.32	19.04	22.92	24.65	58.18
M20	43.61	55.42	1.61	56.36	42.92
M5	56.84	37.91	11.94	43.12	50.04
RF20	57.84	42.01	0.31	42.16	57.62
RF5	54.41	45.11	0.71	45.52	54.10

Note

These proportions were obtained from the analysis of cell‐by‐cell data from the maps of Figure 3, where (B5–B20) Bioclim, (D5–D20) Domain, (G5–G20) Garp, (M5–M20) MaxEnt, (GL5–GL20) GLM, and (RF5–RF20) Random Forest.

Accuracy measure values were higher than would be expected from a null model (Figure 4). We did not find large differences in sensitivity, specificity, AUC, and TSS values obtained from the training and test data. Conversely, we detected differences in under‐ and overprediction (Figure 4). Under‐prediction rates from the training data showed lower values in all methods except DOMAIN, and higher values in overprediction rates than those obtained with the test data. These rates precisely represent the similarity of the models with the ED; BIOCLIM, DOMAIN, and GARP, for example, showed high values of overprediction rates (Figures 3 and 4). On the other hand, RF and MaxEnt presented low values for these rates and their predictions adjusted well with the ED (Figures 3 and 4). The maximum sensitivity value came from DOMAIN (D5–D20), followed by Random Forest (RF5–RF20). DOMAIN, in turn, had the lowest specificity values, while RF presented the highest. BIOCLIM and GARP had the lowest sensitivity values, with high specificity values. This last index was the only accuracy measure with the same values for the training and test data. Maximum TSS and AUC values were obtained in RF, followed by M20, GLM, and M5. TSS showed the highest values in the training data (Figure 4). AUC (ROC curve) was higher than 0.74 in all algorithms. BIOCLIM presented the highest under‐prediction rate, while DOMAIN, GLM5, and RF presented the lowest rate. The highest overprediction rate came from DOMAIN, whereas M20 and RF showed the lowest values. The under‐prediction rate obtained for the training data was between 0 (D5, RF5 and RF20) and 0.0438, with B5 reaching the lowest values. The overprediction rate showed values between 0 and 0.83, with the highest value in D5 (0.8379) and the lowest in RF20 (0), RF5 (0.0014), M20 (0.0109), and GLM20 (0.0624). Once more, D5 reached the highest value (0.403) (Figure 4). We obtained a positive correlation between FGM and the accuracy measures (Table 2) for Specificity, AUC and TSS (*p* < 0.05) (Figures 2 and 4). Therefore, projections of the models with higher ability of discrimination presented greater similarity with the ED of *B. alternatus*.

**Table 2:** Spearman's correlation coefficients between fuzzy global matching and discrimination measures (*p* < 0.05)

Variable I	Variable II	*N*	Spearman	*p*‐Value (*p* < 0.05)
Fuzzy global matching	Sensitivity	12	0.31	0.324
Fuzzy global matching	Specificity	12	0.82	0.001
Fuzzy global matching	Under‐prediction rate	12	−0.31	0.319
Fuzzy global matching	Overprediction rate	12	−0.88	0.00015
Fuzzy global matching	AUC	12	0.89	0.000067
Fuzzy global matching	TSS	12	0.90	0.000052

Note

AUC, area under the curve; TSS, true skill statistic.

## 4 DISCUSSION

Soberón and Peterson (2008) proposed that SDMs find regions that “resemble,” in terms of the layers provided, those areas where occurrence points are located, so the rest of the process is interpretation. Our results show that model predictions recognized correctly some regions inhabited by *B. alternatus*, as is also reported by other studies with different taxa that do not used ED, like in Braunisch et al. (2001); Elith et al. (2009); Tognelli et al. (2006). Although comparisons between model predictions and expert maps with empirical data were barely assessed (Duan et al., 2006; Guisan & Thuiller, 2003), evaluating the performance of the model appears as a good alternative when EDs are available (Peterson et al., 2007). These comparisons become necessary when public health actions such as provision of antiophidic serum, conservation actions, establishment of protected areas, among others, are required (Giraudo, 2012; Mateo et al., 2011). In addition, we found more differences between algorithm predictions than between the environmental predictor sets of 5 and 20 variables within each algorithm, as in Bucklin et al. (1991). We used only environmental variables because climate plays a primary role in shaping species’ distributions and additional predictors have minor effects on the accuracy of SDMs and spatial predictions (Bucklin et al., 1991; Fourcade et al., 2012). Also, Merow et al. (2006) express that insights from ecological theory and knowledge of species can guide which type of variables have to be include in the modeling process.

However, we observed differences within the predictors set only in DOMAIN and MaxEnt, where five variables correctly detected more regions inhabited by *B. alternatus* than 20 in agreement with most of the specific literature (like Wang et al., 1999). The most conservative predictions belonged to BIOCLIM, which did not detect most areas inhabited by *B. alternatus*. In accordance with the works of Elith et al. (2009) and Tognelli et al. (2006), our BIOCLIM predictions achieved low values of precision measures. Conversely, DOMAIN predictions achieved high sensitivity values and the lowest specificity values, as was the case in Tognelli et al. (2006). BIOCLIM presented slightly better values than DOMAIN, like in Graham and Hijmans (2006). GARP, GLM, MaxEnt, and RF predictions more closely resembled the ED of *B. alternatus*, but only RF and M could detect the isolated populations in the Southern distribution of the species.

Genetic Algorithm for Rule‐set Prediction correctly detected almost 50% of the inhabited areas and presented higher values than BIOCLIM and DOMAIN. Random Forest and MaxEnt performed well in all the accuracy measures used. These results are similar to those obtained by Bucklin et al. (1991) and Tognelli et al. (2006). Moreover, they are a good alternative for species with disjunct distributions, as was indicated by Bucklin et al. (1991) and Kesler and Walker (2008). Bucklin et al. (1991) and Duan et al. (2006) found that their better predictions presented high values of AUC and TSS. Our high values of positive correlations between FGM and some accuracy measures such as AUC and TSS showed that the greater discrimination capacity of the model is correlated with a greater similarity between its projections and the ED. AUC always indicated better predictions than a null model, even in projections that under‐ or overpredict the ED of *B. alternatus*, making it impossible to make a decision based on this matrix (see Lobo et al., 2018). TSS was the only helpful accuracy measure to assess the performance of SDMs (Allouche et al., 2006), with the same pattern as FGM. In spite of this, the models that reached the highest FGM values (RF and Maxent 20) under‐ or over‐estimated more than 25% of the ED of *B. alternatus*. These differences could be related to the information given by presence‐only models, providing a suitability gradient of observation of the species but without making a difference between presence/absence or detectability (Guillera‐Arroita et al., 2005). Moreover, such differences could be due to not including in the analysis interspecific interactions and dispersion capacity of the species (Soberón & Peterson, 2008).

Di Cola and Chiaraviglio (2014) predicted high suitability values for *B. alternatus* in the north and center of Misiones, throughout Tucumán and in the east of Salta and Jujuy, while Nori et al. (2001) predicted similar values in the north of Misiones, throughout Buenos Aires and in a disjunct area in Tucumán. Conversely, in our study, model predictions (except DOMAIN and GLM) did not achieve high suitability values for these areas. This may be due to the fact that *B. alternatus* is a species that does not occur in forests (Giraudo, 2018; Scrocchi et al., 2009).

We concluded that certain algorithms, like DOMAIN, produce predictions which are too inclusive, while others present more restrictive predictions, such as BIOCLIM. The rest of the algorithms make under‐ and overpredictions, with RF better resembling the ED of *B. alternatus*. Because of the consistent under‐ or overprediction in the models, our results also confirmed the importance of validating them with independent data or expert opinion. According to Loiselle et al. (2011), failure to include independent model validation may potentially lead to serious errors in conservation decision‐making and planning. These issues need to be further analyzed with others focuses. Recently, advances approaches were developed most of them through the Bayesian approach, that has become a good option to deal when the distribution of the species is modeled using point‐references data due to the ease with which prior information can be incorporated along with the fact that it provides a more realistic and accurate estimation of uncertainty (Dutra Silva, Brito de Azevedo, Bento Elias, & Silva, 2009; Martínez‐Minaya, Cameletti, Conesa, & Pennino, 2016; Rodríguez de Rivera & López‐Quílez, 2017). So, more studies testing and comparing ED, FGM, and Bayesian approach are need it, even if our study was not focuses on recent developed Bayesian approach (see Martínez‐Minaya et al., 2016 for a revision).

Finally, we found that both expert maps with ED and FGM function appear as appropriate tools to complement performance indices used in species distribution modeling since they offer an assessment alternative to compare the characteristics of the predictions when EDs are available.

## CONFLICTS OF INTEREST

The authors have no conflicts of interest to declare.

## AUTHOR CONTRIBUTIONS

JAS conceived the ideas, designed the objectives, analyzed the data, results, figures, and maps and wrote the manuscript. MAC analyzed the data and provided suggestions on manuscript improvement. VA collected the field data and performed the data base, provided information about the study site, made the distribution maps of species, and determined the nodes and track and wrote the manuscript. GB revised the language, wrote the manuscript, and made important contribution on the manuscript discussion. ARG conceived the ideas, collected the data and performed the data base, reported information about the natural history of the species, analyzed the results, wrote the manuscripts and helped with focusing the manuscript.

## DATA ACCESSIBILITY

We included the database of *Bothrops alternatus* in Figshare, under CC0 license (see CC0 in https://knowledge.figshare.com/articles/item/what-is-the-most-appropriate-license-for-my-data).
