# Supplementary material for: Species distribution models and empirical test: Comparing predictions with well‐understood geographical distribution of Bothrops alternatus in Argentina
Source: Ecol Evol. 2018 Oct 2;8(21):10497–509. doi: 10.1002/ece3.4517 (PMC6238127; doi:10.1002/ece3.4517)
Supplement: Supplementary file 1 [file ECE3-8-10497-s001.doc]

Appendix S1

Correlation matrix of 19 climate variables taken from WorldClim (<http://www.worldclim.org/bioclim>), and one topographical variable (altitude) taken from R-package Raster (Hijmans *et al.* 2005; Hijmans *et al.,* 2016) and sets of five variables selected (*) after analysis of correlation (Spearman). Correlation values are given in the lower triangle of the matrix, and the two-tailed probabilities are given in the upper.

| **Argbio** | **alt** | **9** | **8** | **7** | **6** | **5** | **4** | **3** | **2** | **19** | **18** | **17** | **16** | **15** | **14** | **13** | **12** | **11** | **10** | **1** |
| --- | --- | --- | --- | --- | --- | --- | --- | --- | --- | --- | --- | --- | --- | --- | --- | --- | --- | --- | --- | --- |
| **alt*** | 0.00 | 0.08 | 0.39 | 0.00 | 0.00 | 0.81 | 0.00 | 0.00 | 0.00 | 0.00 | 0.00 | 0.00 | 0.00 | 0.00 | 0.00 | 0.00 | 0.00 | 0.04 | 0.96 | 0.67 |
| **9** | -0.10 | 0.00 | 0.00 | 0.00 | 0.00 | 0.00 | 0.00 | 0.00 | 0.17 | 0.21 | 0.00 | 0.03 | 0.00 | 0.00 | 0.00 | 0.00 | 0.00 | 0.00 | 0.00 | 0.00 |
| **8** | 0.05 | 0.84 | 0.00 | 0.00 | 0.00 | 0.00 | 0.00 | 0.00 | 0.00 | 0.00 | 0.00 | 0.00 | 0.00 | 0.00 | 0.00 | 0.00 | 0.33 | 0.00 | 0.00 | 0.00 |
| **7** | 0.46 | -0.56 | -0.19 | 0.00 | 0.00 | 0.94 | 0.00 | 0.00 | 0.00 | 0.00 | 0.00 | 0.00 | 0.00 | 0.00 | 0.00 | 0.00 | 0.00 | 0.00 | 0.00 | 0.00 |
| **6*** | -0.35 | 0.93 | 0.72 | -0.74 | 0.00 | 0.00 | 0.00 | 0.00 | 0.00 | 0.00 | 0.00 | 0.00 | 0.00 | 0.11 | 0.06 | 0.00 | 0.00 | 0.00 | 0.00 | 0.00 |
| **5** | 0.01 | 0.76 | 0.86 | 0.00 | 0.64 | 0.00 | 0.00 | 0.00 | 0.00 | 0.00 | 0.00 | 0.00 | 0.00 | 0.00 | 0.00 | 0.00 | 0.71 | 0.00 | 0.00 | 0.00 |
| **4** | 0.21 | -0.82 | -0.51 | 0.84 | -0.86 | -0.35 | 0.00 | 0.00 | 0.00 | 0.00 | 0.00 | 0.01 | 0.00 | 0.15 | 0.10 | 0.00 | 0.00 | 0.00 | 0.00 | 0.00 |
| **3*** | 0.24 | 0.75 | 0.61 | -0.30 | 0.59 | 0.55 | -0.70 | 0.00 | 0.00 | 0.00 | 0.00 | 0.00 | 0.00 | 0.00 | 0.00 | 0.00 | 0.09 | 0.00 | 0.00 | 0.00 |
| **2** | 0.62 | -0.08 | 0.21 | 0.78 | -0.37 | 0.35 | 0.38 | 0.29 | 0.00 | 0.00 | 0.00 | 0.00 | 0.00 | 0.00 | 0.00 | 0.00 | 0.00 | 0.15 | 0.01 | 0.43 |
| **19** | -0.74 | -0.07 | -0.35 | -0.59 | 0.22 | -0.33 | -0.21 | -0.33 | -0.81 | 0.00 | 0.03 | 0.00 | 0.00 | 0.00 | 0.00 | 0.00 | 0.00 | 0.24 | 0.00 | 0.00 |
| **18*** | -0.20 | 0.76 | 0.55 | -0.62 | 0.76 | 0.40 | -0.77 | 0.64 | -0.26 | 0.12 | 0.00 | 0.06 | 0.00 | 0.00 | 0.18 | 0.00 | 0.00 | 0.00 | 0.00 | 0.00 |
| **17** | -0.75 | -0.12 | -0.39 | -0.54 | 0.17 | -0.35 | -0.15 | -0.37 | -0.80 | 0.99 | 0.11 | 0.00 | 0.00 | 0.00 | 0.00 | 0.00 | 0.00 | 0.04 | 0.00 | 0.00 |
| **16** | -0.33 | 0.72 | 0.46 | -0.72 | 0.77 | 0.31 | -0.78 | 0.53 | -0.42 | 0.32 | 0.96 | 0.30 | 0.00 | 0.80 | 0.00 | 0.00 | 0.00 | 0.00 | 0.00 | 0.00 |
| **15** | 0.62 | 0.38 | 0.62 | 0.34 | 0.09 | 0.50 | -0.08 | 0.52 | 0.68 | -0.90 | 0.21 | -0.91 | 0.01 | 0.00 | 0.00 | 0.44 | 0.00 | 0.00 | 0.00 | 0.00 |
| **14** | -0.73 | -0.18 | -0.44 | -0.50 | 0.11 | -0.40 | -0.09 | -0.41 | -0.78 | 0.98 | 0.08 | 0.99 | 0.27 | -0.91 | 0.00 | 0.00 | 0.00 | 0.00 | 0.00 | 0.00 |
| **13** | -0.36 | 0.66 | 0.42 | -0.72 | 0.74 | 0.26 | -0.73 | 0.43 | -0.49 | 0.37 | 0.91 | 0.36 | 0.97 | -0.05 | 0.33 | 0.00 | 0.00 | 0.00 | 0.00 | 0.00 |
| **12*** | -0.66 | 0.35 | 0.06 | -0.73 | 0.56 | -0.02 | -0.55 | 0.10 | -0.72 | 0.80 | 0.64 | 0.80 | 0.78 | -0.53 | 0.77 | 0.80 | 0.00 | 0.00 | 0.04 | 0.00 |
| **11** | -0.12 | 0.99 | 0.85 | -0.55 | 0.94 | 0.77 | -0.81 | 0.74 | -0.08 | -0.07 | 0.76 | -0.12 | 0.72 | 0.38 | -0.18 | 0.66 | 0.36 | 0.00 | 0.00 | 0.00 |
| **10** | 0.00 | 0.91 | 0.92 | -0.26 | 0.81 | 0.93 | -0.57 | 0.64 | 0.15 | -0.26 | 0.57 | -0.30 | 0.50 | 0.51 | -0.36 | 0.45 | 0.12 | 0.92 | 0.00 | 0.00 |
| **1** | -0.02 | 0.98 | 0.90 | -0.42 | 0.89 | 0.85 | -0.71 | 0.73 | 0.05 | -0.19 | 0.70 | -0.24 | 0.63 | 0.48 | -0.30 | 0.58 | 0.23 | 0.98 | 0.97 | 0.00 |

Legend. Correlation matrix of 19 climate variables and sets of five variables selected (*) after analysis of correlation (Spearman). Where argbioalt (Altitude (m)), argbio1 (Annual Mean Temperature (°C)), argbio2 (Mean Diurnal Range (°C)), argbio3 (Isothermality (°C)), argbio4 (Temperature Seasonality (°C)), argbio5 (Max Temperature of Warmest Month (°C)), argbio6 (Min Temperature of Coldest Month (°C)), argbio7 (Temperature Annual Range (°C)), argbio8 (Mean Temperature of Wettest Quarter (°C)), argbio9 (Mean Temperature of Driest Quarter (°C)), argbio10 (Mean Temperature of Warmest Quarter (°C)), argbio11 (Mean Temperature of Coldest Quarter (°C)), argbio12 (Annual Precipitation (mm)), argbio13 (Precipitation of Wettest Month (mm)), argbio14 (Precipitation of Driest Month (mm)), argbio15 (Precipitation Seasonality (mm)), argbio16 (Precipitation of Wettest Quarter (mm)), argbio17 (Precipitation of Driest Quarter (mm)), argbio18 (Precipitation of Warmest Quarter (mm)) and argbio19 (Precipitation of Coldest Quarter (mm)).
